# Optimal Combination of Non-Invasive Tools for the Early Detection of Potentially Life-Threatening Emergencies in Gynecology

**DOI:** 10.1371/journal.pone.0162301

**Published:** 2016-09-01

**Authors:** Catalina Varas, Marion Ravit, Camille Mimoun, Pierre Panel, Cyrille Huchon, Arnaud Fauconnier

**Affiliations:** 1 EA 7285 Research Unit "Risk and Safety in Clinical Medicine for Women and Perinatal Health", Versailles-Saint-Quentin University (UVSQ), Montigny-le-Bretonneux, France; 2 Department of Gynecology and Obstetrics, Intercommunal Hospital Centre of Poissy-Saint-Germain-en-Laye, Poissy, France; 3 Department of Gynecology and Obstetrics, Mignot Hospital, Le Chesnay, France; West China Second Hospital, Sichuan University, CHINA

## Abstract

**Objectives:**

Potentially life-threatening gynecological emergencies (G-PLEs) are acute pelvic conditions that may spontaneously evolve into a life-threatening situation, or those for which there is a risk of sequelae or death in the absence of prompt diagnosis and treatment. The objective of this study was to identify the best combination of non-invasive diagnostic tools to ensure an accurate diagnosis and timely response when faced with G-PLEs for patients arriving with acute pelvic pain at the Gynecological Emergency Department (ED).

**Methods:**

The data on non-invasive diagnostic tools were sourced from the records of patients presenting at the ED of two hospitals in the Parisian suburbs (France) with acute pelvic pain between September 2006 and April 2008. The medical history of the patients was obtained through a standardized questionnaire completed for a prospective observational study, and missing information was completed with data sourced from the medical forms. Diagnostic tool categories were predefined as a collection of signs or symptoms. We analyzed the association of each sign/symptom with G-PLEs using Pearson’s Chi-Square or Fischer’s exact tests. Symptoms and signs associated with G-PLEs (p-value < 0.20) were subjected to logistic regression to evaluate the diagnostic value of each of the predefined diagnostic tools and in various combinations.

**Results:**

The data of 365 patients with acute pelvic pain were analyzed, of whom 103 were confirmed to have a PLE. We analyzed five diagnostic tools by logistic regression: Triage Process, History-Taking, Physical Examination, Ultrasonography, and Biological Exams. The combination of History-Taking and Ultrasonography had a C-index of 0.83, the highest for a model combining two tools.

**Conclusions:**

The use of a standardized self-assessment questionnaire for history-taking and focal ultrasound examination were found to be the most successful tool combination for the diagnosis of gynecological emergencies in a Gynecological ED. Additional tools, such as physical examination, do not add substantial diagnostic value.

## Introduction

Acute pelvic pain accounts for up to 40% of the visits to gynecological emergency departments (G-EDs) [1] and may indicate a serious condition. Potentially life-threatening gynecological emergencies (G-PLEs) are acute pelvic conditions that may spontaneously evolve into a life-threatening situation. They may also carry a risk of sequelae (organ failure or organ removal) or death in the absence of prompt diagnosis and treatment [2–5].

The most common G-PLEs encountered in G-EDs [3, 6–8] are ruptured ectopic pregnancy [9], adnexal torsion [10], and complicated pelvic inflammatory disease (tubo-ovarian and pyosalpinx) [3, 6–8, 11]. Missing these high-risk conditions may delay treatment that could lead to potentially negative patient outcomes [1, 6]. The early identification of patients with PLEs who require prompt surgical treatment is thus crucial.

In this context, history-taking taking [12, 13], physical examination [12, 13], ultrasonography [14, 15], and laboratory tests [16] are the most appropriate tools. They are currently available in routine practice in most Gynecological EDs. Their diagnostic performance for the identification of patients with G-PLEs has been widely described in the literature [4]. However, the use of these tools in developed countries is highly variable. They can also be used in combination as a diagnostic strategy. For example, obstetrics/gynecology physicians in France routinely perform the initial evaluation of patients seen in G-EDs, including bedside transvaginal ultrasonography, whereas emergency ultrasound is performed in most other developed countries at the request of ED physicians by radiologists or board-certified obstetricians/gynecologists [17]. Most developing countries do not even have an ultrasound in the G-ED [18].

The purpose of the present study was to select the best package of non-invasive diagnostic tools to diagnose PLEs in patients presenting with acute pelvic pain at the G-ED.

## Materials and Methods

### Study design

The study was approved by the French Department of Higher Education and Research (N°06.336) and the French National Committee for Information Technology and Individual Liberties (N°906253). The participants did not directly provide written consent to participate in this study, but accepted to complete a Self-Assessment Questionnaire for Gynecological Emergency (SAQ-GE) accompanied by instructions. This consent procedure was approved by the ethics committees.

Part of the data were collected from a prospective observational multicenter study designed to evaluate the usefulness of a standardized questionnaire for the systematic assessment of the medical history of patients referred for acute pelvic pain to the G-ED [9]. An important limitation of this previous study was that it was based on history-taking only and did not compare this approach with classical diagnostic tools (i.e. clinical and ultrasound examination). For the purpose of the present study, we completed the data using a standardized data collection form for medical records of the patients included in the two main centers to. We collected information on the diagnostic tools used including the triage process, physical examination, ultrasound examination, and biological examination. No new patients were included.

### Study Setting and population

The study included all patients > 18 years of age presenting with acute pelvic pain at G-EDs of the Poissy-St Germain Hospital and Mignot Hospital, Versailles, between September 2006 and April 2008, and hospitalized for any therapeutic intervention or observation. These two hospitals serve a large population in the Parisian suburbs, and have a resident and a senior gynecologist on duty 24 hours a day with day and night access to ultrasound, CT scan, and operating rooms. They also have appropriate written pain management guidelines. In France, obstetrics and gynecology (Ob/Gyn) residents perform the initial evaluation of patients seen in gynecologic EDs, including routine bedside transvaginal sonography. The residents are required to follow a written protocol previously developed for bedside emergency ultrasonography and available online. These guidelines involve the routine recording of four standardized images: i) view of Morison’s pouch with the transabdominal probe; ii) longitudinal view of the uterus to look for the midline stripe indicating an empty uterus; and iii) view of each ovary with the transvaginal probe. One to three additional views may be obtained as dictated by abnormal ultrasound findings (e.g., view of an ectopic gestational sac). The images are stored in the patient’s medical chart, even when no abnormalities are detected [19]. The exclusion criteria were: i) women who had a history of chronic pelvic pain; ii) women presenting with acute pain of an origin other than abdominal and/or pelvic (*i*.*e*. acute breast or vulva conditions); iii) women with an intrauterine pregnancy of greater than 13 weeks gestation.

### Study protocol

A nurse performed a triage process on all patients including: taking a brief history, measuring vital signs (heart rate and arterial pressure [20], and measuring the pain intensity on a self-reported 11-point numerical rating scale (NRS).

All patients had to complete a Self-Assessment Questionnaire for Gynecological Emergency (SAQ-GE) based on history taking. The SAQ-GE was developed for the assessment of acute pelvic pain with a qualitative description, the location and intensity of the pain, and other signs such as vomiting, vaginal discharge, or syncope.

The physical examinations, including palpation of the abdomen and vaginal examination, were performed by the gynecology resident on duty.

Transvaginal ultrasonography (TVUS) was performed using a 3.5–5 MHz transabdominal probe and a 7 MHz transvaginal probe. The residents followed a standardized TVUS protocol including at least four images as described in the previous section. In cases of abnormalities, additional views were performed: a view of the uterine cavity content (*i*.*e*. gestational sac, other abnormality) and a view including any abnormal extra-uterine image (for example, extra-uterine gestational sac, ovarian cyst, etc.) [21].

Biological exams were performed if necessary, and included: a urinary hCG test, and if positive, a serum hCG assay. Blood counts were performed to determine whether the women had anemia based on serum hemoglobin levels [22], infection based on leucocyte counts [20], or inflammation based on C-reactive protein levels [23].

Additional investigations were performed (biological exams, complete ultrasonography by a certified obstetrician/gynecologist, CT scan) if needed. All women received appropriate pain management. This emergency protocol was supervised by a senior gynecologist who decided whether or not to perform emergency laparoscopy based on all of the available data. Patients who did not undergo surgery and received medical treatment were followed until their exams were negative and were classified as non-PLE. For example, women with miscarriage or uncomplicated ectopic pregnancy were followed until their hCG assay was negative.

### Key Outcome Measures

Signs and symptoms were classified as positive or negative using pre-determined information obtained through the analysis of the available literature (Appendix 1). For example, abdominal palpation was considered to be positive when there was guarding, rebound, or mass on abdominal palpation [10, 23]; TVUS was considered to be positive when pelvic fluid reached the uterine corpus or was found around the ovary [13, 22], or in cases of an ovarian cyst larger than 50 mm [24]. The entire description of signs and symptoms used in the study can be found in the appendix.

Signs and symptoms were grouped together into diagnostic tool categories according to the way they were collected (Appendix 1). For example: *Triage* Process was represented by Pulse, SBP, Shock index, and NRS; and *Physical Examination* by rebound tenderness, abdominal guarding, adnexal mass, and adnexal tenderness (Appendix 1).

### Gold standard

For the purpose of the study, G-PLEs were defined as (i) complicated ectopic pregnancy (tubal rupture or active bleeding); (ii) complicated pelvic inflammatory disease (tubo-ovarian abscess or pyosalpinx); (iii) adnexal torsion; (iv) hemoperitoneum exceeding 500 ml of any gynecological origin; (v) appendicitis; and (vi) intestinal obstruction. Non G-PLEs were defined as acute conditions expected to resolve spontaneously or with appropriate medical treatment such as uncomplicated ectopic pregnancy, uncomplicated pelvic inflammatory disease, uncomplicated cyst, myoma, or miscarriage. The reference standard was the visualization of G-PLEs at surgery (laparoscopy or laparotomy) with or without histological confirmation. For the non-G-PLE patients, the diagnosis was performed using either a non-surgical algorithm for early pregnancy complications [25], a non-surgical diagnosis of PID by the non-invasive prediction rule of the Centers for Disease Control and Prevention (CDC) [26], by reference imaging (including reference ultrasound examination or computed tomography), or by direct visualization at surgery. Both centers used similar diagnostic procedures.

### Data Analysis

We first analyzed the diagnostic accuracy of signs/symptoms to discriminate between the two groups (G-PLEs and non- G-PLEs) using Pearson’s Chi-Square or Fischer’s exact test. The diagnostic value of each sign/symptom found to be associated with G-PLEs at a threshold of p < 0.20 was then estimated by calculating the sensitivity (Se), specificity (Sp), positive likelihood ratio (LR+), and negative likelihood ratio (LR-) with a 95% CI.

We constructed different logistic models to evaluate the overall diagnostic value of each of the predefined diagnostic tool categories. First, we included all signs/symptoms which were significantly associated with G-PLEs in the univariate analysis in a logistic regression. Each predefined diagnostic tool category (for example, physical examination) (see appendix 1) was represented by its signs/symptoms (for example, rebound tenderness and guarding) significantly associated with G-PLEs.

Then, we constructed several logistic regressions, including distinct sets of tools, to evaluate the diagnostic value of the tools in combination. For example, we constructed a logistic regression to analyze *Triage Process* alone and another to analyze the diagnostic value of *Triage Process* combined with *Physical Examination*.

In the logistic models, the overall predictive information provided by each tool, and their possible combinations in the model, were expressed by the c-index. The c-index is defined as the proportion of all usable patient pairs in which the predictions and outcomes are concordant, which for a binary logistic regression is equivalent to the area under the receiver operating characteristic curve for a diagnostic tool [27]. Missing data were incorporated as dummy variables [28].

We constructed a forest plot to easily compare the diagnostic value of tools and their possible combinations. This plot presents the c-index and confidence interval for each tool and in combinations of two, three, four, or all, in a graphic manner.

Analyses were carried out using Stata^®^ version 13.0 software (Stata Corp., College Station, Texas, USA.).

## Results

Out of the 460 patients available between September 2006 and April 2008, 46 had one or more exclusion criteria. Among the 414 eligible patients, 49 had missing or incomplete medical records, leaving 365 patients with complete data sets for the statistical analysis; from which 103 had confirmed G-PLEs (28.2%) and 262 other diagnoses (71.8%).

The distribution of each sign/symptom was verified before classification into two classes. No outlier or influential data point was detected.

The diagnostic performance characteristics of the variables (symptoms and signs) associated with G-PLEs is shown in Table 1. All of these variables accounted for more than 5% of events, except for the Systolic Blood Pressure ≤ 90 mmHg, which accounted for less than 3% of patients (8/307). This is an important sign, despite the fact that it is rare, and its specificity was excellent. None of the variables had a sensitivity greater than 90%. Seven variables had a specificity greater than 90%: systolic blood pressure ≤ 90 mmHg, shoulder pain, abdominal guarding, rebound tenderness, fluid in the Morison pouch, pelvic fluid reaching the uterus corpus, and an ovarian cyst larger than 50 mm (Table 1). Pulse, shock index, scapula pain, awakened by pain, pain resistant to drugs, pain when coughing, fainting, syncope, leucorrhea, abnormal vaginal discharge, digital vaginal examination, C-reactive protein, and hemoglobin concentration were not significantly associated with PLEs in the univariate analysis.

**Table 1:** Diagnostic accuracy of selected signs in the univariate analysis with p < 0.20 for the diagnosis of potentially life-threatening gynecological emergencies (G-PLEs).

Diagnostic tools	Patients with the characteristic (365)	Se (%)	Sp (%)	LR +	LR -	p-value*
**Triage process**						
Systolic Blood Pressure ≤ 90 mmHg*	8/307	6.9	99.1	7.59	0.94	0.003
Numerical Rating Scale (NRS) at the worst time >7°	245/358	82.2	37.0	1.30	0.48	0.000
**History-taking**						
History of ectopic pregnancy°	81/362	38.2	83.8	2.37	0.74	0.000
Shoulder pain°	27/347	14.0	94.7	2.66	0.91	0.006
Unbearable pain°	182/339	67.0	51.7	1.39	0.64	0.002
Pain during movement °	243/340	83.5	33.3	1.25	0.49	0.002
Pain on abdominal palpation°	226/335	81.9	38.2	1.32	0.47	<0.001
Vomiting during pain°	84/356	37.0	81.6	2.02	0.77	0.000
**Physical examination**						
Abdominal guarding*^1^	44/365	21.4	91.6	2.54	0.86	0.000
Rebound tenderness*^1^	45/365	18.4	90.1	1.86	0.91	0.069
**Ultrasound signs**						
Fluid in Morrison pouch* ^1^	24/365	16.5	97.3	6.18	0.86	0.000
Pelvic fluid reaching the uterine corpus*^1^	38/365	24.3	95.0	4.89	0.80	0.000
Abnormal adnexal mass*^1^	192/365	81.6	58.8	1.98	0.31	0.000
Mass larger than 50 mm*^1^	40/365	19.4	92.4	2.54	0.87	0.000
**Biological exams**						
Urine hCG test or serum hCG* among patients without known pregnancy	159/250	69.5	39.3	1.14	0.76	0.175
Leucocyte count > 10 G/L*	154/353	56.4	61.5	1.47	0.71	0.002

^1^ Missing data were considered to be normal,

° Prospective Questionnaire evaluation,

* Retrospective Data from record.

Five logistic regression models represent the diagnostic value of the different categories of diagnostic tools: (i) The *Triage Process* model included systolic blood pressure < 90 mmHg and NRS at the worst time > 7; (ii) The *History-taking* model included a history of ectopic pregnancy, shoulder pain, unbearable pain, pain during movement, pain on abdominal palpation, and vomiting during pain; (iii) The *Physical examination* model included abdominal guarding and rebound tenderness; (iv) The *Ultrasonography* model comprised fluid in the Morison pouch, pelvic fluid reaching the uterine corpus, abnormal adnexal mass, and ovarian cyst larger than 50 mm; (v) the *Biological Exams* model was composed of the urine hCG test or serum hCG assay among patients without a known pregnancy and a leucocyte count > 10 G/L. Table 2 presents the c-index for the five logistic regression models and those of the saturated model including the complete combination of all five tools. The saturated model leaving out one tool shows the loss of information after removal of a tool. The highest c-index was obtained for history-taking (0.76) and ultrasonography (0.77). The complete saturated model had a c-index of 0.86. Only the removal of ultrasonography from the saturated model was associated with a significant loss of information (Table 2).

**Table 2:** C-index of non-invasive tools for the diagnosis of potentially life-threatening gynecological emergencies (G-PLEs) using multiple logistic regression.

	C-index of each tool alone [CI 95%]	C-index of the saturated model without the tool [CI 95%]
Triage process	0.61 [0.56; 0.67]	0.84 [0.80; 0.89]
History-taking	0.76 [0.71; 0.82]	0.82 [0.77; 0.86]
Physical examination	0.59 [0.54; 0.64]	0.86 [0.82; 0.90]
Ultrasonography	0.77 [0.72; 0.82]	0.81 [0.76; 0.85]
Biological exams	0.64 [0.57; 0.70]	0.84 [0.80; 0.89]
C-index of saturated model		0.86 [0.82; 0.90]

The C-index indicates the amount of information of a given diagnostic tool. It also indicates the loss of information after removal of a variable from the saturated model.

**Fig 1:**
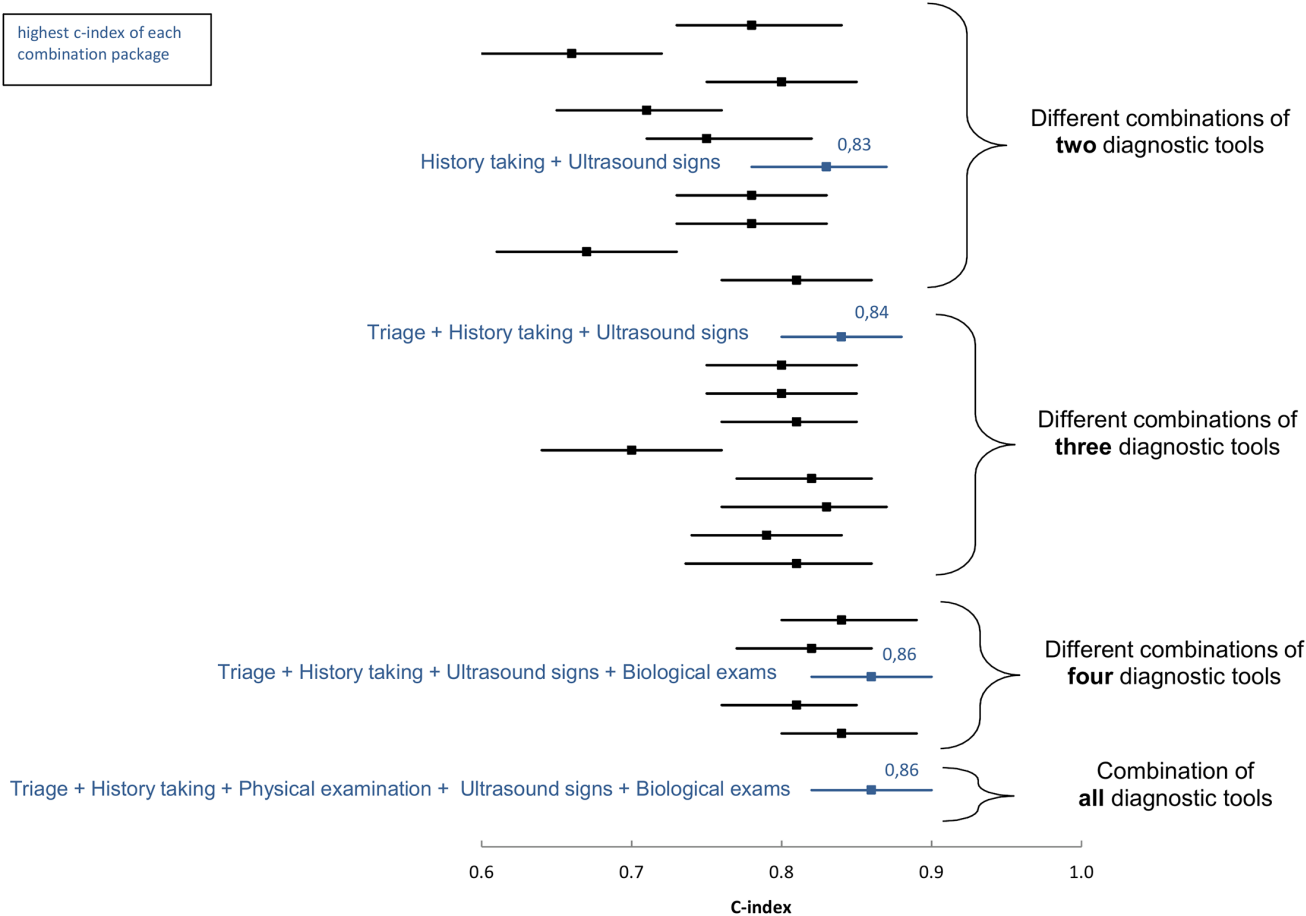
Diagnostic usefulness (represented by the c-index and its 95% confidence interval) of different available diagnostic tools in different combinations.

A forest plot presents the c-index and confidence interval for all possible tool combinations (Fig 1). Among all logistic models, the combination of history-taking and ultrasound signs had a c-index of 0.83. This is the two-tool model with the highest c-index. The best model with three diagnostic tools was obtained by incorporating triage, history-taking, and ultrasonography and had a c-index of 0.84. The best logistic model with four diagnostic tools was obtained by including biological exams with the last three and had a c-index of 0.86.

## Discussion

History-taking, with the use of a standardized questionnaire, and ultrasonography, based on a standardized image acquisition procedure, provide sufficient diagnostic information for the detection of G-PLEs among women consulting for acute pelvic pain at the G-ED. The diagnostic value of adding other tools was not substantial. Moreover, our study does not support the traditional role of physical examination, including abdominal and vaginal digital examination, in the diagnostic procedure for acute gynecological emergency conditions.

The originality of this work has been to consider the most representative high-risk conditions encountered in a G-ED that need to be promptly managed to avoid complications with long-term impacts on the health and well-being of women, rather than a single gynecological condition. Such a global view of high-risk situations is essential for determining the best diagnostic approaches and identifying the facilities required by gynecological emergency services [4].

To test the diagnostic utility of a given diagnostic tool as a whole (i.e clinical examination or ultrasonography, for example), the different available tools were modeled by combining signs and/or symptoms which demonstrated diagnostic utility, instead of just studying sign by sign [25]. This allowed us to study the clinical utility of different sets of diagnostic tools from the point of view of the organization of available care and resources [6, 29] and not from the traditional medical point of view.

Clinical examination, including abdominal examination and digital vaginal examination, is traditionally performed as a part of the diagnostic procedures for gynecologic emergencies in most care models in OECD countries. In one prospective study, clinical examination was found to be very useful for the diagnosis of ectopic pregnancy in an emergency care model with limited access to ultrasound [30]. In contrast, routine performance of digital vaginal examination was found to be completely useless for the diagnosis of ectopic pregnancy in a model of care based on unlimited use of ultrasound and serum hCG assays [25]. With the introduction of ultrasound scans in the 1990’s, there has been little interest in studying clinical examination in the literature and recent well-designed studies evaluating the clinical examination in the context of gynecological emergencies are lacking [4]. This is likely explained by the limited additional diagnostic information provided by vaginal examination relative to that obtained by bedside emergency ultrasonography [31], as well as the potential hazards of this examination [16, 32] due to the lack of reproducibility and inter-observer variability.

Our results show that emergency bedside ultrasonography may be the cornerstone of the diagnosis of G-PLEs. This is in complete agreement with the results of a systematic review specifically designed to identify non-invasive tools for the diagnosis of G-PLE. Indeed, based on 20 studies, transvaginal ultrasound was the individual diagnostic tool with the best performance for the diagnosis of G-PLEs [4]. However, these studies lack any information about the qualifications and experience of the clinicians performing the ultrasound, contrary to the present study. Furthermore, these studies did not provide any comparison with other diagnostic tools that would place ultrasonography examination in the context of the entire diagnostic work-up process.

Medical history taking has been found to be useful in the context of acute pelvic or abdominal pain in women in some retrospective studies [12, 13, 33–35]. However, items collated by the physician may be misinterpreted as they are not based on standardized questions found in self-assessment questionnaires that are directly understandable by patients. We thus developed a standardized self-assessment questionnaire that does not require intervention from healthcare professionals. This questionnaire has already proven useful for triaging most G-PLEs including ruptured ectopic pregnancy [24], adnexal torsion [10], and pelvic inflammatory disease [36]. The present study shows that the assessment of medical history by standardized questionnaire may not only be useful for triage purposes, but may also be a significant component of the entire diagnostic process as it brings independent and important information to the medical staff in charge of the diagnosis of gynecological emergencies.

Vital signs, including the measurement of heart rate, blood pressure, and pain intensity using a NRS, were found to be predictors of G-PLEs. However, the independent contribution to the diagnosis was low relative to those brought by ultrasonography or medical history taking. Nonetheless, vital signs, including the shock index calculation, have already proven to be an important aide in detecting instability which frequently occurs in G-PLEs [4]. The shock index is an easily obtained calculation based on physiological variables that are measured in every patient and has been shown to correlate well with early acute hypovolemia [37]. Due to the design of the present study, the rate of instability was low. We nonetheless recommend systematically performing vital sign measurements on arrival to the ED.

In most developed countries, emergency ultrasound is performed at the request of ED physicians by radiologists or board-certified obstetricians/gynecologists [17, 38]. Patients in most developing countries do not have access to ultrasound in the G-ED [18]. In contrast, in the French model of gynecological emergency care, women with acute pelvic pain can have direct evaluation by ultrasound in G-EDs, to which all women have free access. Obstetrics/gynecology residents perform the initial evaluation of patients seen in G-EDs, including bedside transvaginal ultrasonography. Our study suggests that ultrasound facilities should be available around the clock for female patients presenting with acute pelvic pain for better detection of G-PLEs. Indeed, the availability of transvaginal ultrasound at the initial assessment of gynecological emergencies decreases the time to patient care and treatment, and reduces unnecessary admissions.

Another issue is potential variability in the training level of the resident that may slightly skew data. However, this variability is low because Ob/Gyn residents training in France systematically receive basic sonographic training. This training allows all residents to perform standardized sonography dedicated specifically for gynecologic emergencies [19].

The training and skills of the practitioner who perform the ultrasonography (US) is an important consideration. The examination is likely to be more accurate when performed by a physician with board certification in gynecological ultrasound examination, on a dedicated machine, than when performed bedside in an emergency context by a non-certified examiner. Most of the US examinations in the present study were performed by non-certified gynecological residents with the use of an appropriate teaching model [19]. A study performed in a general academic ED, with an active US training program for residents, found bedside US to be potentially useful for the diagnosis and management of ectopic pregnancy [39]. It will be important in the future to evaluate teaching models and standardized methods for the implementation of bedside US examinations to improve the competence of non-specialized practitioners in the performance of emergency ultrasound examinations. It may be suitable to implement this teaching process in non-gynecological care models.

Another element is the self-assessment questionnaire that we have developed. It can be immediately given to patients presenting with acute pelvic pain on admission to general EDs by non-clinicians. Implementation of this diagnostic method could be generalized by scaling up, useful in crowded EDs. The self-administered questionnaire can be filled out manually, but with the advent of new technologies, alternative methods of completing it can be considered, such as a host terminal or touch pads. Collection of the SAQ-GE, associated with the collection of other data, would then be instantly processed by a computer and its results would predict patients who belong to a risk group for specific diseases. Combining this information with that obtained by bedside ultrasound examination provided by a non-certified examiner would be a realistic way to improve access of G-PLEs to specialized centers in developing countries. Efficient educational training tools are available for learning how to perform standardized bedside sonographic examinations as a first-line investigation procedure for gynecologic emergency departments [19]. We suggest that implementation of this training process would make it possible for these scans to be performed by anyone involved in gynecologic emergency management (*i*.*e*. ED physicians, family medicine physicians, midwives, and advanced nurse practitioners).

### Limitations

One limitation of our study is related to our definition of G-PLEs. From the medical and pathophysiological point of view, the different conditions that encompass G-PLEs are different entities, but from the point of view of quality of care, they can be viewed as near-miss cases in the G-ED. The main objective of gynecological emergency care departments is to identify high risk patients whose conditions may rapidly deteriorate and pose a potential threat to their life or fertility [4]. Healthcare services must have efficient screening processes for these patients. Any deficiency in this respect may have a major impact by delaying therapeutic intervention. Our definition of G-PLEs is consistent with the clinical reality of patients with gynecological emergencies at risk of severe complications due to diagnostic delays. For example, it was recently suggested that the association between severe maternal morbidity in ectopic pregnancies may be related to the quality of care [6]. From the perspective of quality of care and safety, G-PLEs can be viewed as near-miss cases, and can thus be informative about obstacles that had to be overcome after the onset of an acute complication.

Another limitation is that we included all patients who consulted in G-EDs for acute pelvic pain and were hospitalized. This recruitment strategy is likely to have resulted in a referral bias [40] as it is likely that a large proportion of the hospitalized patients had an abnormal ultrasound image or clinical examination. Patients with acute pelvic pain who were not hospitalized may have had G-PLEs but were misdiagnosed (*i*.*e*. no abnormal criteria were revealed by any examination). However, we believe the risk of missing a surgical emergency among patients who leave the G-ED is very low. Patients with undiagnosed G-PLEs would experience severe complications and severe, persistent pain, and would have eventually returned to our G-EDs which serve a vast geographic area.

A final limitation is that we analyzed a small number of patients (365). However, our study allowed us to define hypotheses that we will test in a larger cohort, URGO, composed of all women who consulted in G-EDs for acute pelvic pain in 21 French hospital centers in the period from March 9 to April 13, 2015.

## Conclusions

The use of a standardized self-assessment questionnaire and focal ultrasonography, including TVUS by non-specialized examiners, was sufficient for the diagnosis of PLEs in G-EDs. Our study does not support the traditional role of physical examination, including abdominal and vaginal digital examination, in the diagnostic procedure for acute gynecological emergency conditions. The diagnostic methods and resources used in emergency departments require further exploration. Further studies are needed to test the performance of this care model in various situations, in particular in the context of low medical ressources.

## Supporting Information

S1 Table Diagnostic tools: definition of signs according to the available literature. (DOCX)

S1 File PLEs data anonym. (XLS)
