# Supplementary material for: Optimal Combination of Non-Invasive Tools for the Early Detection of Potentially Life-Threatening Emergencies in Gynecology
Source: PLoS One. 2016 Sep 1;11(9):e0162301. doi: 10.1371/journal.pone.0162301 (PMC5008751; doi:10.1371/journal.pone.0162301)
Supplement: S1 Table — (DOCX) [file pone.0162301.s001.docx]

**S1 Table: Diagnostic tools: definition of signs according to the available literature.**

| **Tools** | **Signs** | **Cut-off** | **Study** | **Sources** |
| --- | --- | --- | --- | --- |
| **Triage process** |  |  |  |  |
|  | Pulse | ≥ 110 b/min | Fauconnier 2007, Popowski 2012 | B |
|  | Systolic Blood Pressure (SBP) | ≤ 90 mmHg | Fauconnier 2007 | B |
|  | Shock index (Pulse/SBP) | ≥ 0.85  ≥ 0.81 | Birkhahn 2002/ Jaramillo 2011 | B |
|  | Numerical Rating Scale (NRS) at the worst time | > 7 | Popowski 2012, Huchon 2014 | A |
| **History-taking** |  |  |  |  |
|  | History of ectopic pregnancy | yes |  | A |
|  | Scapula pain | yes | Hirata 1991, Fauconnier 2003 | A |
|  | Shoulder pain | yes | Huchon 2012 | A |
|  | Awakened by pain | yes | Bouquier 2014 | A |
|  | Pain resistant to drugs | yes |  | A |
|  | Unbearable pain | yes |  | A |
|  | Pain when coughing | yes | Bouquier 2014 | A |
|  | Pain during movement | yes | Huchon 2012, Popowski 2012 | A |
|  | Pain on abdominal palpation | yes | Bouquier 2014 | A |
|  | Fainting | yes | Hirata 1991 | A |
|  | Syncope | yes | Fauconnier 2003, Huchon 2012 | B |
|  | Vomiting during pain | yes | Huchon 2012 | A |
|  | leucorrhea | yes | RPC 2012, Bouquier 2014 | A |
|  | Abnormal vaginal discharge | yes | RPC 2012, Bouquier 2014 | A |
| **Physical examination** |  |  |  |  |
| Abdominal palpation | Rebound tenderness | present | Mol 1999, Dart 1999 Fauconnier 2003, Popowski 2012 | B |
|  | Abdominal guarding | present | Dart 1999, Fauconnier 2003 | B |
| Digital vaginal examination | Adnexal mass | present | Mol 1999, Fauconnier 2007, Qasi 2010, Popowski 2012 | B |
|  | Adnexal tenderness | present | Stovall 1990, Eschenbach 1997, Buckley 1998 | B |
| **Ultrasound signs** |  |  |  |  |
|  |  |  |  |  |
| Abdominal | Fluid in Morison pouch | present | Moore 2007, Popowski 2012, Toret Labeuw 2013 | B |
| Vaginal | Pelvic fluid | present | Mol 1998, Fauconnier 2007, Popowski 2012 | B |
|  | Pelvic fluid | ≥ uterine isthmus  ≥ uterine corpus  ≥ uterine fundus | Mol 1998, Fauconnier 2007, Popowski 2012 | B |
|  | Fluid around the ovary | present | Fauconnier 2012, Toret Labeuw 2013 | B |
|  | Abnormal adnexal mass | present | Stovall 1990, Buckley 1998, Huchon 2010 | B |
|  | Ovarian Cyst | ≥ 50mm | Huchon 2010 | B |
| **Biological exams** |  |  |  |  |
|  | Urine hCG test | Positif | Fauconnier 2013, Toret Labeuw 2013 | B |
|  | Serum hCG | ≥ 10 mIU/mL | Mol 1998, Qi 2012 | B |
|  | C-reactive protein | ≥ 6 mg/L | Kahn 2005, Kim 2014 | B |
|  | Hemoglobin concentration | ≤ 10 g/dl | Mol 1999, Fauconnier 2007, Popowski 2012 | B |
|  | Leucocyte count | ≥ 10 G/L | Johansson 2007 | B |

A: Prospective Questionnaire evaluation, B: Retrospective Data from record

Triage

History-taking

Physical examination

Ultrasound signs

Biological exam

Figure 1: Diagnostic value (C-index) of tools combination for predicting potentially life-threatening emergencies (PLTEs)

**Each tool**
